# Supplementary material for: Coexistence of Myelin Oligodendrocyte Glycoprotein Immunoglobulin G and Neuronal or Glial Antibodies in the Central Nervous System: A Systematic Review
Source: Brain Sci. 2022 Jul 27;12(8):995. doi: 10.3390/brainsci12080995 (PMC9405704; doi:10.3390/brainsci12080995)
Supplement: Supplementary file 1 [file brainsci-12-00995-s001.zip › brainsci-1823322-supplementary.pdf]

(((((coexist*[Title/Abstract]) OR (dual positive[Title/Abstract])) OR (double positive[Title/Abstract])) OR (triple positive[Title/Abstract])) OR (overlap[Title/Abstract])) AND ((("Myelin-Oligodendrocyte Glycoprotein"[Mesh]) OR ("Oligodendrocyte-Myelin Glycoprotein"[Mesh])) OR (MOG[Title/Abstract]))
